# Energy expenditure and slow-wave sleep in runners: Focusing on reproductive function, chronic training, and sex

**DOI:** 10.1016/j.isci.2024.111717

**Published:** 2025-01-03

**Authors:** Akiko Uchizawa, Haruka Osumi, Simeng Zhang, Katsuhiko Yajima, Airi Funayama, Emi Kondo, Yoko Suzuki, Yoshiaki Tanaka, Insung Park, Yasushi Enomoto, Naomi Omi, Kumpei Tokuyama, Hiroyuki Sagayama

**Affiliations:** 1 Institute of Health and Sport Sciences, University of Tsukuba, Ibaraki, Japan; 2 Advanced Research Initiative for Human High Performance (ARIHHP), University of Tsukuba, Ibaraki, Japan; 3 Graduate School of Comprehensive Human Science, University of Tsukuba, Ibaraki, Japan; 4 International Institute for Integrative Sleep Medicine (WPI-IIIS), University of Tsukuba, Ibaraki, Japan; 5 Faculty of Pharmaceutical Sciences, Josai University, Saitama, Japan

**Keywords:** Kinesiology, Clinical neuroscience

## Abstract

This comparative study focused on chronic exercise training, menstrual cycle, menstruation, and sex related with slow-wave sleep and energy expenditure (EE) during sleep in runners and controls. Participants included 26 highly trained runners (9 males, 8 amenorrheic females, and 9 menstruating females) and 20 controls (10 males and 10 menstruating females) aged 21 ± 2 years. Sleeping metabolic rate and EE during slow-wave sleep were 2.7% and 3.9% higher in the luteal than the follicular phase for female runners. Significant interactions were found between the 8-h time course adjusted EE and menstruation, exercise training, and sex. Sleep stage durations did not differ between groups. Interestingly, amenorrheic runners did not suppress sleeping and overnight metabolic rates, suggesting that EE in sleep may not be a causative factor for amenorrhea in runners. This study highlights the complex relationship between chronic exercise, menstrual cycle, and sex on EE during sleep.

## Highlights

• SMR and EE during SWS differed between menstrual phases in female runners
• Menstrual cycle did not affect RMR, SMR, and OMR in female controls
• Menstruation, chronic exercise, and sex influenced EE fluctuations during sleep
• Amenorrheic runners with PAL ≈2.0 did not show suppressed RMR, SMR, and OMR

## Graphical abstract

**Figure:**
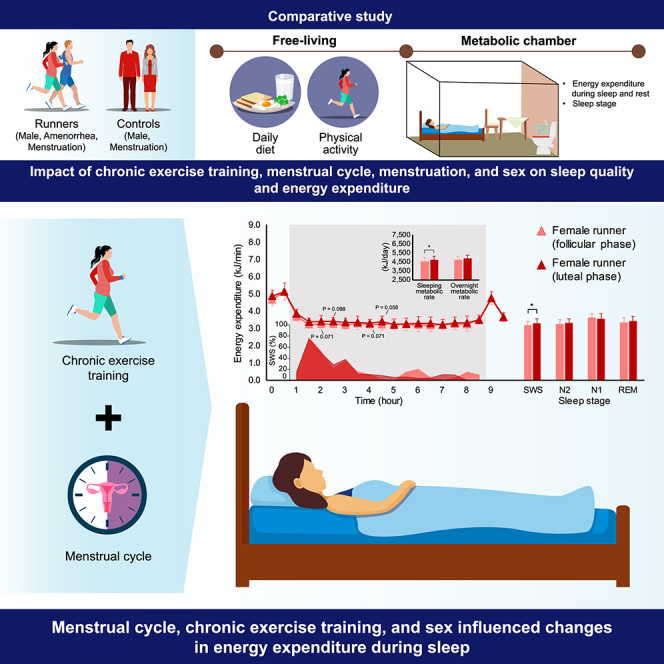

## Teaser

Kinesiology; Clinical nutrition

## Introduction

Long-distance runners compete in sports where weight control is important. They often attempt to maintain a low body weight to optimize their time-based performance.^1^ Several factors, including diet and training, are involved in weight control, particularly in long-distance runners who experience high exercise-induced energy expenditure (EE).^2^ Athletes with less than optimal energy intake relative to exercise EE are at an increased risk of the female athlete triad and relative energy deficiency in sport, which include low energy availability (EA), amenorrhea, and low bone density or osteoporosis.^3^^,^^4^ These syndromes are particularly prevalent in athletes who maintain a low body weight over a long period, such as long-distance runners and aesthetic sports athletes, and are triggered by chronically reduced EA.^5^ EA is predominantly utilized for essential survival functions, such as cellular maintenance and thermoregulation. When EA is low over a long period, luteinizing hormone pulse and frequency may become suppressed,^6^ increasing the risk of menstrual disorders.^7^ Consequently, the energy used for processes that are unnecessary for survival, such as growth and reproduction, is reduced,^8^ and the resting metabolic rate (RMR) may decrease. The ratio of measured-to-estimated RMR calculated from body composition has been used to categorize women as energy deficient.^9^ Sleep patterns can also influence weight control, as an extended sleep duration may reduce energy intake, resulting in a negative energy balance.^10^ Humans spend approximately one-third of each day sleeping, which is crucial for recovery and immune responses in athletes, thus serving essential survival functions. The sleeping metabolic rate (SMR) and the overnight metabolic rate (OMR) represent 90–95% of the RMR.^11^^,^^12^ The SMR and OMR, which are calculated as the mean over an extended duration, are unaffected by temporal arousal, thus being more reproducible and stable than the basal metabolic rate or RMR.^11^^,^^13^

The proportion of slow-wave sleep (SWS) throughout the night is inversely related to the energy balance, suggesting its importance in regulating energy expenditure.^14^ SWS is also associated with the secretion of numerous hormones, including growth hormone, prolactin, cortisol, aldosterone, and melatonin,^15^^,^^16^^,^^17^^,^^18^ which contribute to physical growth and recovery. SWS and sleep efficiency were identified as the strongest predictors of the sleep quality index,^19^ and a relationship between the OMR and the ratio of SWS plus rapid eye movement (REM) sleep to the total sleep time has been reported.^20^ Additionally, recent studies have investigated the link between SWS and sleep quality, highlighting the potential impact of SWS.^21^ Furthermore, previous studies have consistently reported that the SMR was higher in the luteal phase than that in the follicular phase in non-athletes,^22^^,^^23^ indicating menstrual cycle effects on overnight EE,^24^ thus contributing to the SMR. In a previous systematic review, both acute and regular physical activity (once per week or more) were found to increase the duration of SWS, with regular physical activity not chronic exercise such as athletes, showing a larger effect size than acute exercise.^25^ Differences in EE between sleep stages have also been observed, with SWS exhibiting the lowest EE.^26^^,^^27^ However, the complex relationships between menstruation, SWS, and chronic exercise, particularly regarding EE fluctuations during sleep in young individuals with long-term training experience, remain unclear. Thus, menstrual function, chronic exercise training, and SWS may have a combined effect on EE.

We hypothesized that first, the SMR and OMR of runners may be suppressed by chronic exercise training, which influences SWS, especially reflected as an increase in SWS. Second, SWS function may be influenced by differences in the SMR or OMR during the menstrual cycle. Third, SWS influenced by chronic exercise training and reproductive function may drive fluctuations in EE during sleep. Therefore, we aimed to evaluate the influence of chronic exercise training, the menstrual cycle, menstruation, and sex on the SMR, OMR, and successive changes in EE during sleep. Moreover, we compared the various combinations of chronic exercise training, menstrual function, and sex in terms of sleep architecture and EE at each sleep stage that influenced EE during sleep.

## Results

### Characteristics

The physical characteristics of the participants are presented in Table 1. The physical activity level (PAL) of the runners was 2.05 ± 0.24 (range 1.67–2.56), which was significantly higher than that of controls, which was 1.67 ± 0.13 (range 1.50–2.04) (*p* < 0.05). Fat-free mass (FFM) was higher in males than that in females, with no difference between groups of female runners. The ratio of measured-to-estimated RMR was above the cutoff value of 0.94 for all groups. The female athlete triad risk score was significantly higher in amenorrheic runners than that in controls. Additionally, seven of the eight amenorrheic runners had experienced irregular menstruation or amenorrhea for over two years (average, 37.5 ± 14.6 months; range, 15–55 months).

**Table 1:** Characteristics of study participants

	*Runner*												*Control*								
	Female									Male (*n* = 9)			Female						Male (*n* = 10)		
	Amenorrhea (*n* = 8)			Follicular phase (*n* = 9)			Luteal phase (*n* = 7)						Follicular phase (*n* = 10)			Luteal phase (*n* = 10)					
Age, year	19.6	±	1.5^∗^	19.2	±	0.7^∗^	19.3	±	0.8	21.3	±	1.3	22.6 ± 2.3						21.6	±	2.1
Body weight, kg	46.7	±	5.8^†‡^	48.6	±	4.4^†‡^	49.3	±	4.7	60.1	±	6.5	54.4	±	8.7^‡^	54.8	±	8.4	65.2	±	9.0
Height, cm	156.5	±	5.3^†‡^	160.9	±	4.6^†‡^	161.2	±	4.3	173.3	±	8.0	162.3 ± 7.2^†‡^						175.3	±	4.5
BMI, kg/m^2^	19.1	±	2.2	18.8	±	1.4	18.9	±	1.5	20.0	±	1.0	20.6	±	2.2	20.7	±	2.1	21.2	±	2.6
FM, kg	8.5	±	3.5^∗^	9.1	±	2.1^∗^	9.4	±	2.4	4.8	±	1.1	15.1	±	5.3^†^	15.4	±	5.0	10.6	±	4.4^†^
Percent FM, %	17.6	±	5.7^∗†^	18.5	±	3.2^∗†^	18.9	±	3.5	8.0	±	1.4	27.3	±	5.3^†‡^	27.7	±	4.8	15.7	±	4.7^†^
FFM, kg	38.2	±	3.3^†‡^	39.6	±	2.8^†‡^	39.9	±	2.9	55.3	±	5.6	39.3	±	4.5^†‡^	39.4	±	4.4	54.7	±	5.2
Bone mineral density *Z* score	−0.54	±	1.14	−0.23	±	1.60	0.13	±	1.62	0.51	±	1.23	−0.51 ± 0.99						−0.29	±	0.92
mRMR, kJ/day	5123	±	417	5018	±	545	5110	±	346	7193	±	852	5177	±	668	5370	±	765	6975	±	964
RMR ratio	1.08	±	0.07	1.05	±	0.13	1.06	±	0.08	1.16	±	0.11	1.09	±	0.12	1.13	±	0.10	1.10	±	0.14
Energy availability, kcal/kg FFM/day	37.0	±	10.9	36.2	±	10.5	36.2	±	10.7	24.5	±	10.0^∗^	43.6	±	4.6^†^	42.9	±	9.0	36.3	±	9.4
Core body temperature during sleep, °C	36.50	±	0.26	36.36	±	0.57	36.65	±	0.33	36.57	±	0.24	36.61	±	0.25	36.87	±	0.26^∗^	36.34	±	0.37
PAL	2.07	±	0.27^∗‡^	1.97	±	0.14^‡^	2.01	±	0.10	2.12	±	0.30	1.70	±	0.17^†^	1.60	±	0.11	1.65	±	0.09^†^
Menarche age, year	13.9	±	1.0^∗^	13.6	±	2.5	13.9	±	2.6	–			11.9 ± 0.7						–		
Female athlete triad risk score	4.4	±	2.5^∗^	2.2	±	2.7	2.4	±	2.9	–			0.5 ± 0.5						–		

Physical characteristics in runners and healthy young adults. Data are expressed as mean ± SD. The characteristics of participants were analyzed using one-way ANOVA between five groups (amenorrheic runner, and female runner and female control in the follicular phase, male runner, and male control). Comparisons of the intra-individual mRMR, core body temperature and PAL between menstrual cycles were examined using a Student’s paired t test. BMI, body mass index; FM, fat mass; FFM, fat-free mass; mRMR, measured resting metabolic rate; PAL, physical activity level. ^∗^*p* < 0.05 vs. female control in the follicular phase; ^†^*p* < 0.05 vs. male runner, ^‡^*p* < 0.05 vs. male of control.

### Correlation of resting, sleeping and overnight metabolic rates, and the ratio of measured-to-estimated energy metabolic rate

A strong correlation was observed between measured SMR (mSMR) and measured RMR (mRMR) (*r* = 0.966; *p* < 0.01), measured OMR (mOMR) and mRMR (*r* = 0.969; *p* < 0.01), as well as mSMR and mOMR (*r* = 0.997; *p* < 0.01). The SMR ratio of female runners and controls during the follicular phase was significantly lower than that in male runners (Table 2).

**Table 2:** Absolute values of energy expenditure and ratio for measured-to-predicted energy expenditure during sleeping

	*Runner*												*Control*								
	Female									Male (*n* = 9)			Female						Male (*n* = 10)		
	Amenorrhea (*n* = 8)			Follicular phase (*n* = 9)			Luteal phase (*n* = 7)						Follicular phase (*n* = 10)			Luteal phase (*n* = 10)					
mSMR, kJ/day	4692	±	534	4516	±	399	4673	±	452	6453	±	754	4478	±	729	4705	±	633	6202	±	890
SMR ratio	0.99	±	0.10	0.94	±	0.08^†^	0.97	±	0.10^∗^	1.10	±	0.07	0.93	±	0.11^†^	0.97	±	0.07	1.05	±	0.13
mOMR, kJ/day	4851	±	570	4686	±	374	4853	±	408	6709	±	749	4638	±	716	4871	±	630	6404	±	959
OMR ratio	1.04	±	0.09	0.99	±	0.08	1.01	±	0.09	1.11	±	0.07	0.95	±	0.10^†^	0.99	±	0.05	1.04	±	0.13

The ratio of measured-to-predicted energy expenditure in runners and healthy young adults. Data are expressed as mean ± SD. There were nine participants in the female control group for the ratios of sleeping and overnight metabolic rates because one adult had missing data for body composition using DXA. The ratios of measured-to-predicted energy expenditure were analyzed using one-way ANOVA between five groups (amenorrheic runner, and female runner and female control in follicular phase, male runner, and male control). Intra-individual comparisons between menstrual cycles were examined using a Student’s paired t test. mSMR, measured sleeping metabolic rate; mOMR, measured overnight metabolic rate; FFM, fat-free mass; FM, fat mass. ^∗^*p* < 0.05 vs. female runner in follicular phase, ^†^*p* < 0.05 vs. male runner.

### Sleep architecture and energy expenditure during sleep

Sleep architecture variables such as REM sleep, non-REM sleep stage 1 (N1), non-REM sleep stage 2 (N2), and SWS were compared between groups based on the duration of sleep phases (Tables 3 and S1). The duration of N1 for menstruation runners in the follicular phase was shorter than that for male controls (*p* = 0.011).

**Figure 1:**
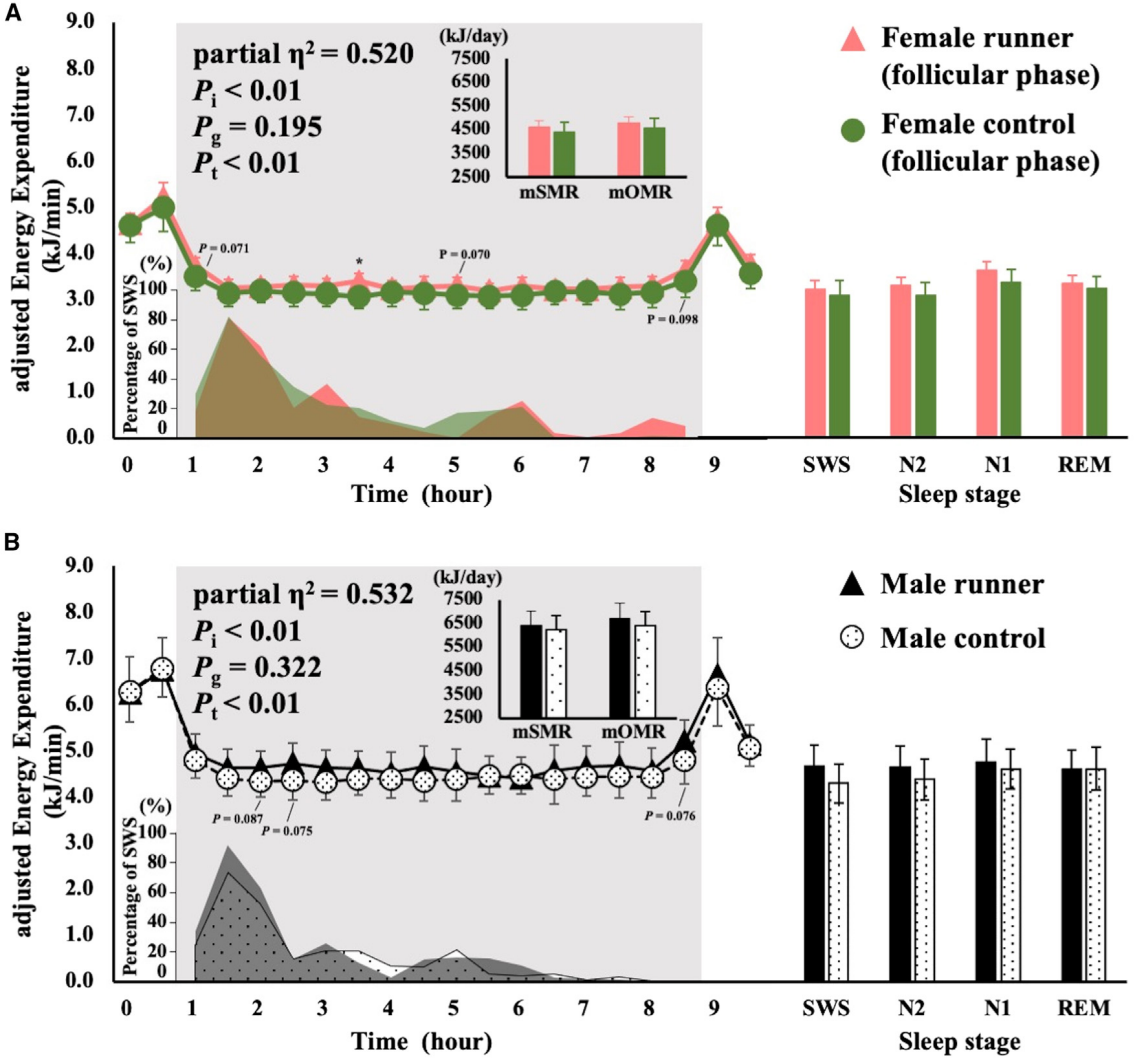
Comparison between chronic exercise in terms of overnight EE, percentage of SWS time, and average EE at sleep stages SMR and OMR are shown in the upper left panel; the change in overnight EE is shown in the center of the left panel; the percentage of SWS time is shown in the bottom left panel; the average EE at each sleep stage is shown in the right panel. EE is shown as mean ± SD. Differences in EE between the groups were analyzed using ANCOVA with FFM and FM as covariates. The interaction for the time course of EE was analyzed using two-way ANCOVA for mixed designs with FFM and FM as covariates. *p* values of two-way ANCOVA are shown as *P*_i_ for the interaction, *P*_g_ for the main effect of group, and *P*_t_ for the main effect of time. Runners are indicated by triangles; controls are indicated by circles. ∗*p* < 0.05. SMR, sleeping metabolic rate; OMR, overnight metabolic rate; SWS, slow-wave sleep; N2, non-rapid eye movement sleep stage 2; N1, non-rapid eye movement sleep stage 1; REM, rapid eye movement sleep. (A) Comparison between menstruating runners and female controls in the follicular phase (pink indicates menstruating runners; green indicates female controls). (B) Comparison between male runners and male controls (black indicates male runners; black dots with black borders indicates male controls).

**Figure 2:**
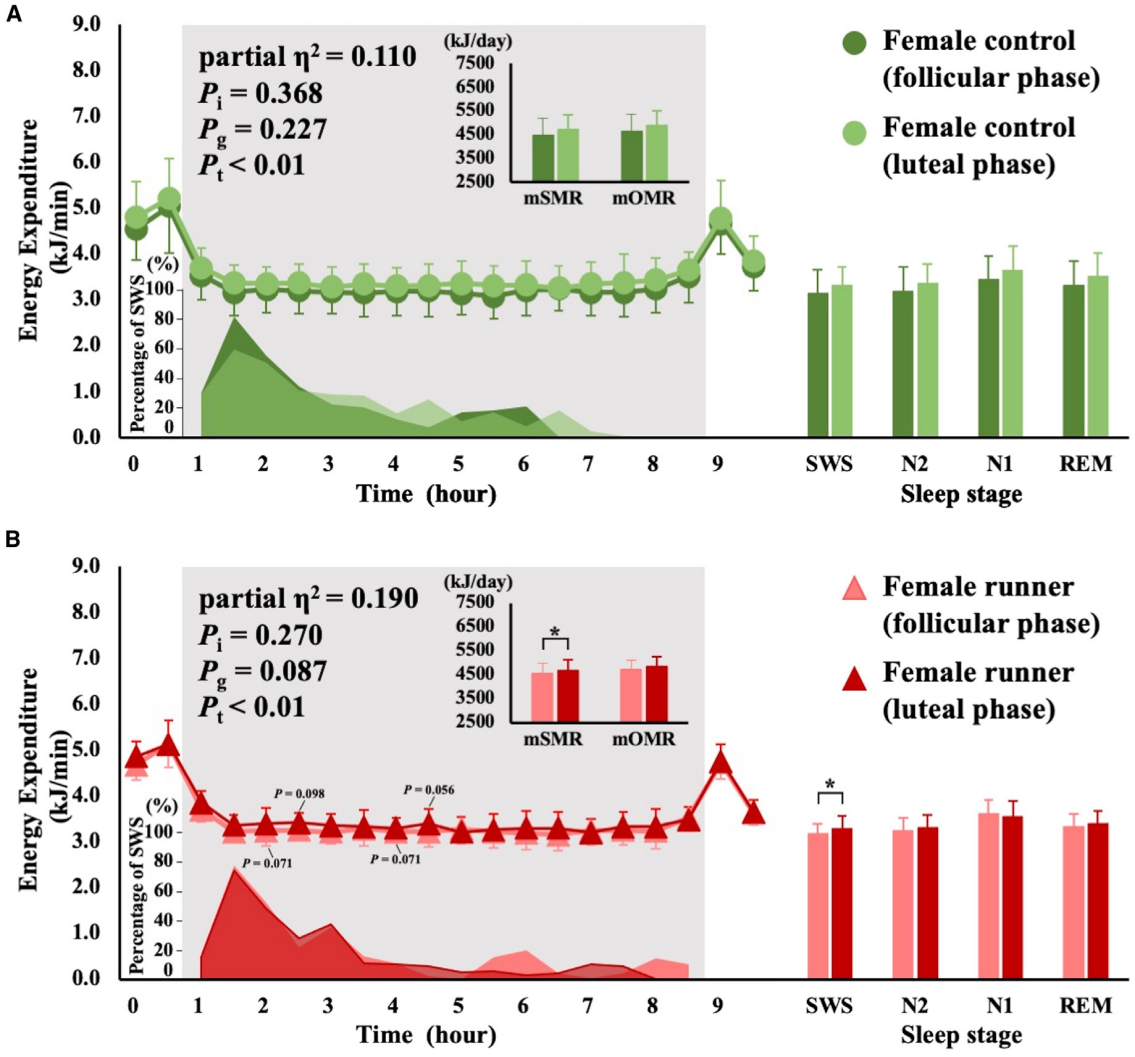
Comparison between the menstrual cycle in terms of overnight EE, percentage of SWS time, and average EE at sleep stages SMR and OMR are shown in the upper left panel; the change in overnight EE is shown in the center of the left panel; the percentage of SWS time is shown in the bottom left panel; the average EE at each sleep stage is shown in the right panel. EE is shown as mean ± SD. The differences in EE between the follicular and luteal phases were analyzed using Student’s paired *t*-tests. The interaction for the time course of EE was analyzed using two-way ANOVA for within-subject designs. *p* values of two-way ANOVA are shown as *P*_i_ for the interaction, *P*_g_ for the main effect of group, and *P*_t_ for the main effect of time. Controls are indicated by circles; runners are indicated by triangles. ∗*p* < 0.05. SMR, sleeping metabolic rate; OMR, overnight metabolic rate; SWS, slow-wave sleep; N2, non-rapid eye movement sleep stage 2; N1, non-rapid eye movement sleep stage 1; REM, rapid eye movement sleep. (A) Comparison between the follicular and luteal phases in female controls (green indicates the follicular phase; light green indicates the luteal phase). (B) Comparison between the follicular and luteal phases in female runners (pink indicates the follicular phase; red indicates the luteal phase).

**Figure 3:**
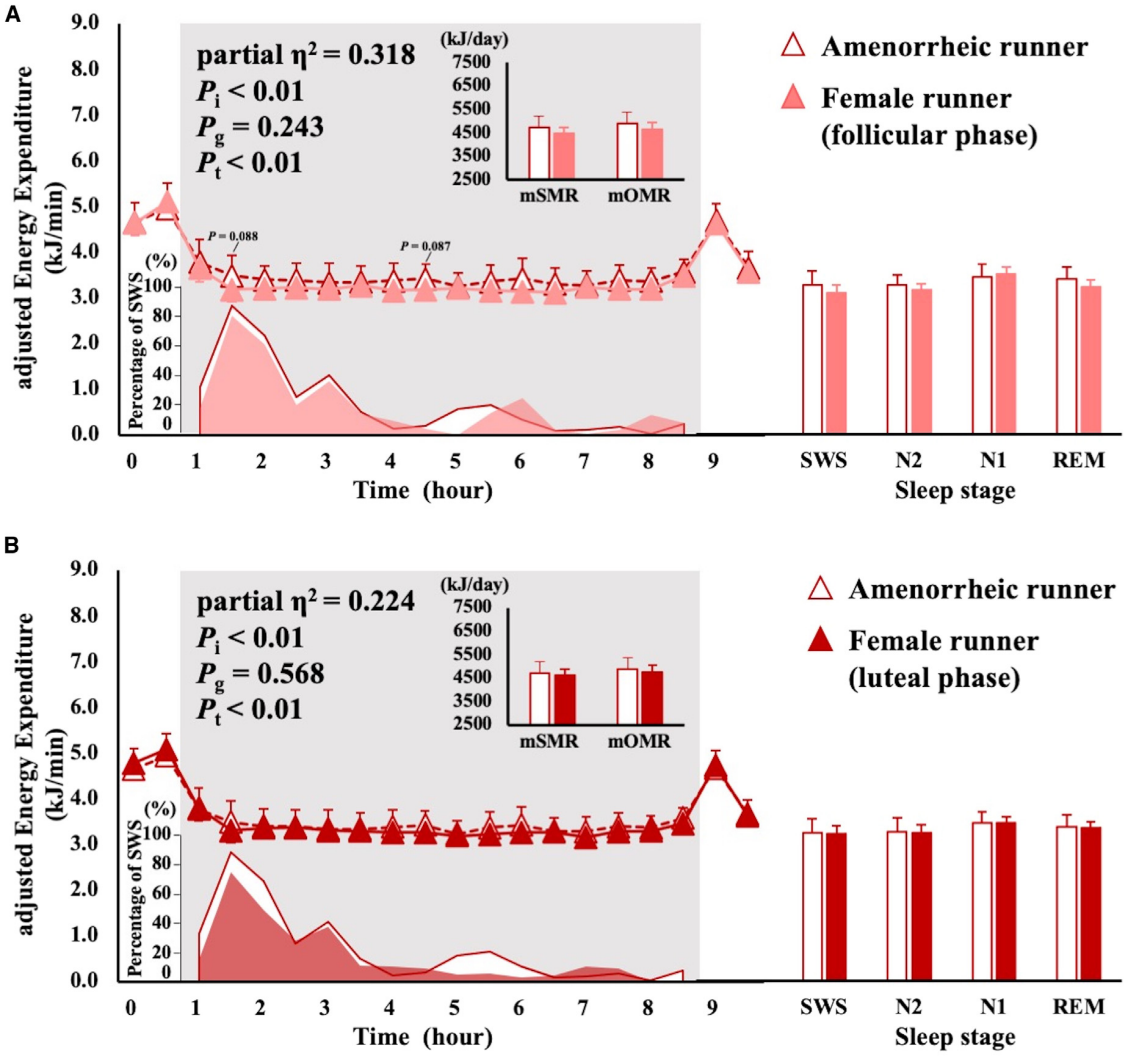
Comparison of menstruation in terms of overnight EE, percentage of SWS time, and average EE at sleep stages SMR and OMR are shown in the upper left panel; the change in overnight EE is shown in the center of the left panel; the percentage of SWS time is shown in the bottom left panel; the average EE at each sleep stage is shown in the right panel. EE is shown as mean ± SD. Differences in EE between the groups were analyzed using ANCOVA with FFM and FM as covariates. The interaction for the time course of EE was analyzed using two-way ANCOVA for mixed designs with FFM and FM as covariates. *p* values of two-way ANCOVA are shown as *P*_i_ for the interaction, *P*_g_ for the main effect of group, and *P*_t_ for the main effect of time. Runners are indicated by triangles. SMR, sleeping metabolic rate; OMR, overnight metabolic rate; SWS, slow-wave sleep; N2, non-rapid eye movement sleep stage 2; N1, non-rapid eye movement sleep stage 1; REM, rapid eye movement sleep. (A) Comparison between amenorrheic runners and menstruating runners in the follicular phase (white with red borders indicates amenorrheic runners; pink indicates menstruating runners in the follicular phase). (B) Comparison between amenorrheic runners and menstruating runners in the luteal phase (white with red borders indicates amenorrheic runners, and red indicates menstruating runners in the luteal phase).

**Figure 4:**
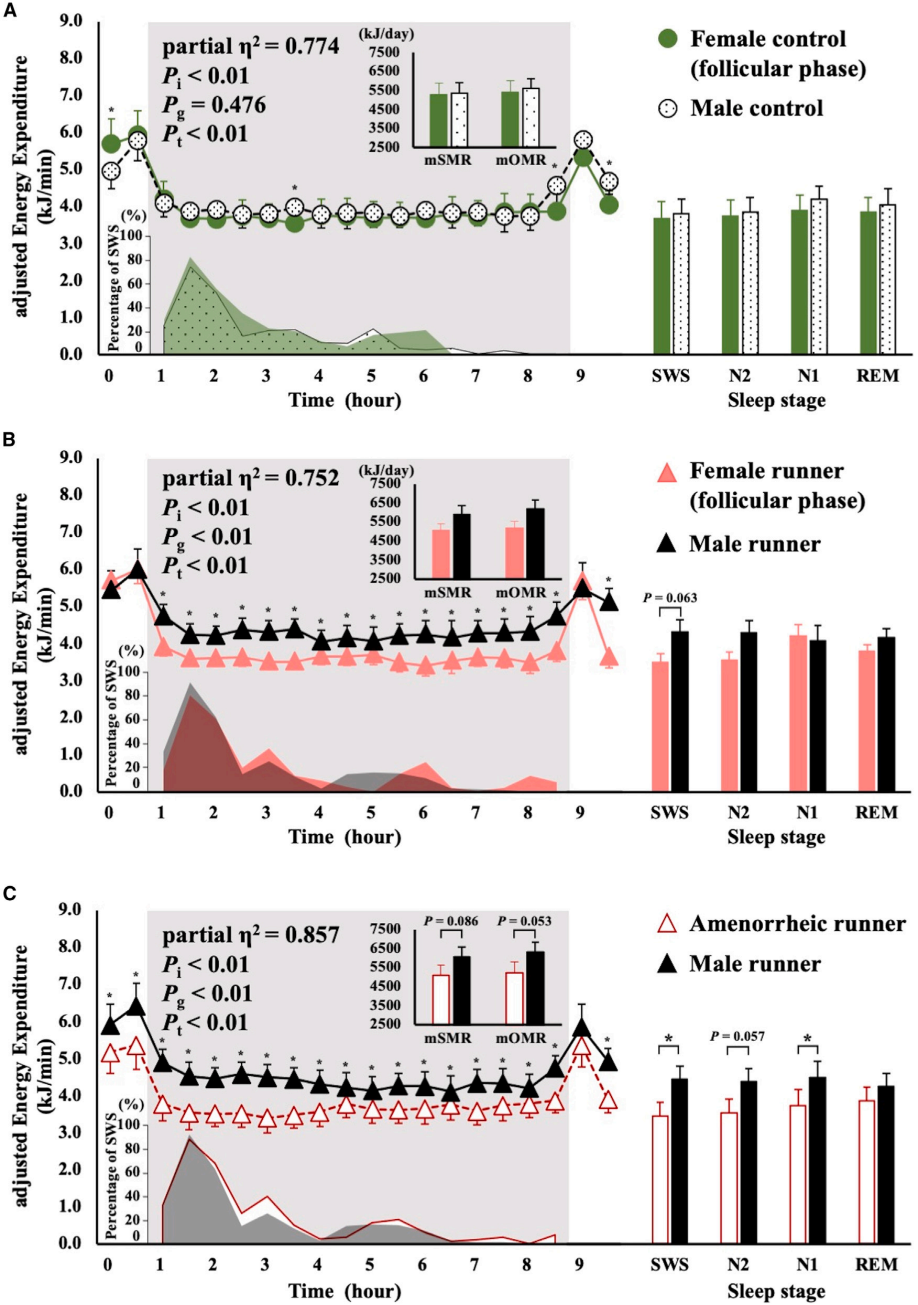
Comparison between sexes regarding overnight EE, percentage of SWS time, and average EE at sleep stages SMR and OMR are shown in the upper left panel; the change in overnight EE is shown in the center of the left panel; the percentage of SWS time is shown in the bottom left panel; the average EE at each sleep stage is shown in the right panel. EE is shown as mean ± SD. Differences in EE between the groups were analyzed using ANCOVA with FFM and FM as covariates. The interaction for the time course of EE was analyzed using two-way ANCOVA for mixed designs with FFM and FM as covariates. *p* values of two-way ANCOVA are shown as *P*_i_ for the interaction, *P*_g_ for the main effect of group, and *P*_t_ for the main effect of time. Controls are indicated by circles; runners are indicated by triangles. ∗*p* < 0.05. SMR, sleeping metabolic rate; OMR, overnight metabolic rate; SWS, slow-wave sleep; N2, non-rapid eye movement sleep stage 2; N1, non-rapid eye movement sleep stage 1; REM, rapid eye movement sleep. (A) Comparison between female controls in the follicular phase and male controls (green indicates female controls in the follicular phase; black dots with black borders indicates male controls). (B) Comparison between menstruating runners in the follicular phase and male runners (pink indicates menstruating runners in the follicular phase; black indicates male runners). (C) Comparison between amenorrheic runners and male runners (white with red borders indicates amenorrheic runners; black indicates male runners).

The time course of EE during sleep, calculated every 30 min, was evaluated to assess the effects of time and other study parameters (menstrual cycle, with or without menstruation, with or without exercise training, and sex; Figures 1, 2, 3, and 4, left panel), and the EE at each sleep stage was compared between the groups (Figures 1, 2, 3, and 4, right panel). There was a significant interaction between time course and chronic exercise training, whereas the EE during each sleep stage adjusted by fat mass (FM) and FFM did not differ between the groups (Figures 1A and 1B). The female controls exhibited a gradual decrease in SWS after the first peak, whereas female runners had three SWS peaks. Adjusted EE at 180 min after bedtime was significantly higher in female runners than in controls (*p* = 0.006; Figure 1A).

The results of the comparison between menstrual phases are shown in Figure 2. There was no interaction between time course and menstrual phase concerning EE in the runner and control groups. The mSMR was significantly higher in the luteal phase than that in the follicular phase for runners (*n* = 7, follicular phase: 4552 ± 425 kJ/day, luteal phase: 4673 ± 452 kJ/day, *p* = 0.035). EE during SWS was 3.9% higher in the luteal phase than that in the follicular phase in runners (*p* = 0.038). The interaction between time course and menstruation was statistically significant, with no difference in EE during each sleep stage between groups (Figures 3A and 3B). Female runners had three SWS peaks that occurred the earliest in amenorrhea, followed by the follicular and luteal phases.

An interaction between time course and sex was observed for all combinations of runners and controls (Figures 4A–4C), with the main effect of sex in runners (menstruating; *p* < 0.01, amenorrhea; *p* = 0.001). Furthermore, adjusted EE was significantly higher for male runners than that for female runners for all time courses during sleep (Figures 4B and 4C). There was no difference in adjusted EE of sleep stages in controls, while adjusted EE in SWS (*p* = 0.021) and N1 (*p* = 0.045) was 44.5% and 39.8% higher for male runners than that for amenorrheic runners, respectively.

## Discussion

This study investigated differences in EE during sleep and various aspects of sleep architecture, focusing on SWS, which affects many physiological systems and is considered an essential component of SWS. We analyzed these factors in relation to menstrual cycle, menstruation, chronic exercise training, and sex.

### Chronic exercise training on EE during sleep and SWS

This study examined differences in EE during sleep associated with chronic exercise training, accounting for menstrual phases and sex. Our results in runners are similar to those of Schulz et al. in that RMR and SMR adjusted for EI and FFM, as a chronic training effect, did not differ between well-trained endurance athletes (runners, cyclists, and triathletes) and untrained adults.^28^ Although there was no difference in the duration of each sleep stage (Tables 3 and S1), SWS timing differed with and without chronic exercise training (Figure 1). A previous study has shown that the exercise timing before bedtime affects sleep quality in non-athletes, regardless of their daily physical activity.^29^ Furthermore, SWS in athletes was shorter on sedentary than that on exercise days.^30^ All participants in this study avoided training for 24 h before bedtime on the measurement day to remove the effects of acute exercise on EE^31^; thus, we likely found no significant difference in SWS, unlike that reported in previous studies. We observed significant interactions between the time course during sleep and chronic exercise training (Figure 1). Chronic exercise training influenced changes in EE during sleep, although it did not influence the duration of each sleep stage without acute training on the measurement day. Exercise can alter plasma melatonin levels, which is one of the main hormones that controls the sleep-wake cycle.^32^ Hence, fluctuations in EE during sleep may be related to melatonin secretion. The effect of exercise on melatonin and fluctuations in EE during sleep needs to be further explored. Endogenous melatonin analyses could provide more comprehensive insights in this respect.

The impact of differences in exercise type on SWS and sleep quality has been highlighted in previous research. According to the meta-analysis review by Kredlow et al., acute exercise shows a moderate beneficial effect on SWS with cycling, whereas such benefits are not observed with acute running.^29^ Regarding regular exercise, mind/body exercises (e.g., yoga, tai chi) are reported to have a significant positive impact on sleep quality, and similar benefits are also noted for traditional exercises (e.g., walking, aerobic exercise). However, no significant differences between these two types of regular exercise have been identified.^29^ To the best of our knowledge, the present study is the first to reveal fluctuations in SWS and metabolism during sleep in runners undergoing chronic exercise training.

#### Menstrual cycle on EE during sleep and SWS

Previous studies have reported that SMR is slightly higher in the luteal phase, with a different calculation range during sleep; however, the method for calculating SMR and the timing for measurement differed from those of the current study.^22^^,^^23^ As described by Benton et al.,^33^ the menstrual cycle has a small yet significant effect on RMR; however, there was no significant effect in more recent studies published after 2000. Thus, RMR and SMR may be measured with higher accuracy given improved measurement techniques, and the results may differ from those of previous studies. Therefore, the current study examined the effects of the menstrual cycle on SMR for a minimum of 3 h and overnight for 8 h using a metabolic chamber, and only the mSMR of female runners in the luteal phase was higher than that in the follicular phase. The duration of each sleep stage was similar in the follicular and luteal phases for both the runners and controls (Table 3), and the results were similar to those of previous studies.^24^ Exercise increases the subsequent EE due to excess post-exercise consumption,^31^; thus, all participants avoided training for 24 h before bedtime on the day of the metabolic measurements in this study. The PAL of female runners in each menstrual phase was comparable (Table 1). Hence, we considered it unlikely that excess post-exercise consumption affected the EE differences during SWS between menstrual phases in runners.

**Table 3:** Architecture of sleep-related variables

	*Runner*												*Control*								
	Female									Male (*n* = 9)			Female						Male (*n* = 10)		
	Amenorrhea (*n* = 7)			Follicular phase (*n* = 9)			Luteal phase (*n* = 7)						Follicular phase (*n* = 9)			Luteal phase (*n* = 10)					
Sleep period time, min	473.0	±	6.3	471.2	±	7.0	468.6	±	8.8	476.2	±	3.4	470.5	±	9.4	471.8	±	9.1	471.9	±	9.6
Total sleep time, min	456.4	±	21.4	454.8	±	16.4	457.3	±	13.9	465.9	±	4.7	451.5	±	25.8	448.2	±	23.7	460.6	±	14.1
Wake after sleep onset, min	16.6	±	15.7	16.4	±	10.0	11.3	±	8.4	10.3	±	4.6	19.0	±	21.5	23.6	±	24.2	17.6	±	16.8
REM, min	83.3	±	19.1	79.6	±	18.3	79.6	±	19.6	96.8	±	17.4	84.5	±	12.9	74.3	±	14.0	82.5	±	16.8
N1, min	34.8	±	8.1	24.3	±	10.4^∗^	41.2	±	12.7	38.0	±	11.0	37.8	±	29.3	45.1	±	26.1	52.4	±	17.0
N2, min	233.7	±	35.7	256.6	±	25.0	256.1	±	13.5	237.6	±	24.1	232.1	±	34.3	231.8	±	28.6	238.6	±	23.7
SWS, min	104.6	±	25.4	94.3	±	23.3	80.3	±	7.9	93.5	±	27.5	97.1	±	26.5	97.2	±	31.3	80.9	±	25.0
Ratio of SWS+REM, %	41.1	±	7.3	38.1	±	7.0	34.9	±	3.6	40.8	±	6.8	40.3	±	5.7	38.4	±	8.4	35.5	±	6.2
Sleep efficiency, %	96.5	±	3.4	96.5	±	2.2	97.6	±	1.8	97.8	±	1.0	96.0	±	4.6	95.0	±	5.1	97.6	±	1.6

Architecture of sleep-related variables during the time in bed in runners and healthy young adults. Data are expressed as mean ± SD. Sleep period time was defined as the time between sleep onset and last awaking, including wake time after sleep onset. Total sleep time was defined as the time between sleep onset and last waking, excluding wake time after sleep onset. Ratio of SWS + REM (%) was defined as the percentage of REM sleep and SWS to the total sleep time. Sleep efficiency (%) was defined as the percentage of total sleep time to the sleep period time. There were seven amenorrheic participants and nine female controls in the follicular phase because one adult had missing data for sleep architecture. The sleep architectures were analyzed using one-way ANOVA between five groups (amenorrheic runner, and female runner and female control in the follicular phase, male runner, and male control). Intra-individual comparisons between menstrual cycles were examined using a Student’s paired t test. REM, rapid eye movement sleep; N1, non-rapid eye movement sleep stage 1; N2, non-rapid eye movement sleep stage 2; SWS, slow-wave sleep. ^∗^*p* < 0.05 vs. male control.

No significant differences were observed in the sleep architecture during the three minimum EE consecutive hours utilized for calculating mSMR between menstrual phases. EE during SWS in the luteal phase was higher than that during the follicular phase, and the mSMR onset time overlapped with the timing of the third peak of SWS (Figures 2 and S1). Furthermore, the difference in mSMR between menstrual phases was in line with the difference in EE during SWS. Therefore, we considered that SWS, particularly SWS during the time without the masking effect from physical activity before bedtime, could potentially account for a difference in mSMR; hence, the third peak of SWS may hold physiological significance. SWS underlies the immune-boosting effects of sleep,^34^ and tumor necrosis factor α (TNFα) and interleukin-1β (IL-1β) production is increased in the luteal phase compared to that in the follicular phase.^35^^,^^36^ Immune function is also affected by exercise time and intensity.^37^ Therefore, the difference in EE during SWS and mSMR between the menstrual phases of female runners with normal menstruation may have been influenced by immune function,^38^ which is affected by chronic exercise and sex hormones. The combination of the menstrual cycle and chronic exercise training may underpin the differences in the mSMR of female runners during the menstrual cycle (Table S2). We speculate that SWS is related to SMR and EE fluctuations during sleep^39^; however, it is unclear which factor has a prior effect on EE during sleep or sleep architecture.

### EE during sleep and SWS upon suppression of menstruation

Amenorrheic runners did not exhibit a suppressed mRMR (Table 1), mSMR, or mOMR (Table 2). Hence, we considered that amenorrhea may not have been induced by energy deficiency in this study. Indeed, mSMR, mOMR, and EE in SWS were similar between amenorrheic and menstruating runners in both menstrual phases (Figure 3). In a previous study, values between 2.2 and 2.5 were proposed as the upper PAL limit humans can maintain for a prolonged period of time, in which physical activity above a PAL value of 2.5 entails the risk of a constrained total EE.^40^^,^^41^ In this study, the PAL for amenorrheic runners, excluding two runners, was less than 2.2 (range 1.67–2.52). Hence, EE adjusted for FM and FFM in SWS could also be affected by factors other than the menstrual cycle and exercise training. Amenorrheic runners may experience negative effects on their reproductive function due to other factors, independent of EE suppression. For example, the human hypothalamic-pituitary-ovarian system regulates sex hormone secretion, and physical stress can disrupt sex hormone control, resulting in decreased estradiol secretion.^42^^,^^43^ Physical stress not only stimulates the sympathetic nervous system, leading to increased EE, but may also regulate ovarian hormone secretion via sympathetic brainstem pathways under dysfunctional hypothalamic hormonal control.^44^ Therefore, amenorrhea in runners may have been induced by physical stress in the current study.

## Sex on EE during sleep and SWS

We not only compared menstrual females and males in controls and runners but also amenorrheic runners and male runners to determine whether suppression of reproductive function further affects sex differences in EE and sleep quality. There were significant differences and trends of sex in EE for SWS in runners (Figures 4B and 4C). The combination of sex and chronic exercise training may drive the differences in EE during sleep and EE during SWS (Figure 4), similar to the effects of the menstrual cycle in runners. There were significant differences in EE during SWS between amenorrheic and male runners. Male runners and amenorrheic runners underwent exercise training without a menstrual cycle. However, amenorrhea indicates suppression of reproductive function in female runners, which is not the case in male runners. We speculated that the suppression of reproductive function is a relevant factor, and that long-term suppression may have a greater impact on EE changes than exercise training or sex.

Male runners had similar EE in all sleep stages, and EE at each sleep stage may have increased in non-REM sleep or decreased in REM sleep (Figure S2). Jurov et al. reported that male endurance athletes experience decreases in testosterone and triiodothyronine when their EA is 9–25 kcal/kg FFM/day, although short-term decreases in EA did not decrease the RMR ratio.^45^ Thus, the approximate 25 kcal/kg FFM/day EA for male runners in the current study could have affected EE between sleep stages; however, the underlying cause remains unclear. SWS modulates endocrine release and inhibits the human hypothalamic-pituitary-ovarian system. Consequently, plasma cortisol, adrenaline, and noradrenaline levels decrease, while growth hormone, prolactin, and melatonin levels increase.^46^^,^^47^ Growth hormones and cortisol play important roles in glucose metabolism, while SWS is associated with decreased brain glucose metabolism.^48^ Thus, sex-based differences in glucose metabolism may have affected EE, and the differences between runners based on sex may arise in relation to physiological functions not directly linked to reproductive function. However, EE cannot be explained by the effects of reproductive suppression alone, as energy metabolism is a complicated process resulting from the intricately interconnected relationships between reproductive function, the immune system, and growth factors. Further studies are warranted to determine whether reproductive function is suppressed in male runners.

Chronic exercise training may influence how SWS is expressed and, consequently, the changes in EE during sleep, although it does not affect the duration of SWS. In addition, the combination of menstrual cycle and chronic exercise training may drive differences in EE during SWS throughout the menstrual cycle. EE during SWS that occurs close to arousal may drive the difference in SMR during the menstrual cycle. Furthermore, the combination of sex and chronic exercise training may drive the differences in EE during sleep and SWS. Therefore, the first factor influencing the SWS may be chronic exercise training, followed by menstrual cycle and reproductive suppression. It would be interesting to investigate whether these differences between the groups also influence the physiological functions of SWS. Taken together, while several questions remain unanswered, our study demonstrates that differences in menstrual function (menstruation, menstrual cycle), chronic exercise training, and sex contribute to different EE phenomena during sleep, all of which may occur in relation to SWS.

### Limitations of the study

This study has some limitations. Meal content was not completely standardized among participants to avoid intervention to their energy balance in daily life. However, we standardized it to the extent possible by ensuring that the same food was consumed twice and by strictly standardizing the finishing time for dinner. Moreover, we calculated the energy intake,^49^ and there was no difference between groups. Dietary-induced thermogenesis almost disappears within 6 h,^50^^,^^51^^,^^52^ and the dinner timing was controlled on the day of EE measurement. mRMR and mOMR were measured at least 13 h and 5 h after a meal, respectively. This study mainly focused on the SMR, which was analyzed based on the mean of the three consecutive hours with the lowest EE, and the average mSMR start time initiated more than 6 h after dinner (Figure S1). Additionally, we confirmed that energy intake from dinner on the day of EE measurement and one-week diets did not differ between the follicular and luteal phases or between runners and controls. Therefore, the effect of diet on EE was deemed negligible. Second, considering unconfirmed ovulation, there is a potential uncertainty regarding the accurate delineation of the menstrual phase, particularly in characterizing the luteal phase. However, we addressed this uncertainty by analyzing the average menstrual cycles over one year and ensuring they did not significantly deviate from the typical menstrual cycle patterns. Notably, in previous studies employing similar methods, we observed menstrual variations in estrogen and progesterone levels during sleep.^24^ To address this limitation, we also measured core body temperature in the metabolic chamber (Table 1). We confirmed that the absence of a menstrual period for over a year in amenorrheic runners allows for a reasonable evaluation of the impact of menstruation on EE during sleep.

We further evaluated the relationship between metabolism and sleep quality utilizing sleep metrics [sleep efficiency, SWS, REM, and (SWS+REM)/total sleep time] introduced in previous studies.^19^^,^^53^^,^^54^ Specifically, the ratio of SWS and REM to the total sleep time has been identified as an indicator of “quality sleep” in studies employing metabolic chambers to measure SMR^20^ and was thus incorporated into our analysis. However, no standardized criteria for objectively defining sleep quality have been reported to date. Consequently, the sleep metrics utilized in this study may not comprehensively reflect relationships with metabolism. Notably, the paucity of previous studies examining the association between SWS, metabolism, and athletic performance limits the ability to draw comprehensive conclusions. Thus, while our findings suggest a relationship between sleep quality and metabolism, their interpretation necessitates caution, and further investigations are required to validate these results.

## Resource availability

### Lead contact

Further information and requests for resources and reagents should be directed to and will be fulfilled by the lead contact, Hiroyuki Sagayama (sagayama.hiroyuki.ka@u.tsukuba.ac.jp).

### Materials availability

This study did not generate new unique reagents.

### Data and code availability

The data that support the findings of this study are available on request from the corresponding author upon reasonable request. The data are not publicly available due to privacy or ethical restrictions.

## Acknowledgments

We wish to thank the volunteers who participated in this study. We appreciate the technical support of Fuji Medical Science Co. (Chiba, Japan).Funding sources: this work was supported by the University of Tsukuba Basic Research Support Program Type S to H.S.; the 10.13039/501100001691JSPS KAKEN to A.U. (22J11154), 10.13039/100010868H.S. (20K19563, 23H03279, 23KK0177) and 10.13039/100016772K.T. (20H04120), and JSC high-performance center Total Conditioning Research Project to N.O.

## Author contributions

A.U., H.O., S.Z., K.T., O.N., and H.S.: study conception and design; A.U., H.O., S.Z., A.F., E.K., Y.T., I.P., and Y.E.: conduct of experiments; A.U., A.F., K.Y., Y.S., and H.S.: data analysis; A.U., E.K., K.T., and H.S.: interpretation of experimental results; A.U. and H.S.: preparation of illustrations; A.U. and H.S.: drafting of the manuscript; A.U., Y.S., Y.T., N.O., K.T., and H.S.: manuscript editing and revising. All authors read and approved the final version of the manuscript.

## Declaration of interests

The authors declare no conflict of interest.

## STAR★Methods

### Key resources table

**Table:** 

REAGENT or RESOURCE	SOURCE	IDENTIFIER
**Software and algorithms**		

Microsoft EXCEL	Microsoft 365	https://www.microsoft.com/ja-jp/microsoft-365
IBM SPSS Statistics 28	IBM SPSS software platform	https://www.ibm.com/spss
G∗Power 3.1.9.7	HHU, Heinrich Heine Universität Düsseldorf	https://www.hhu.de/

### Experimental model and study participant details

#### Study participants

Fifty-two volunteers were recruited for this study. Six were excluded based on the following exclusion criteria and reasons: awake for more than one-third of the sleeping time, or the respiratory exchange ratio exceeded 1.00 during sleep due to malfunction.^55^ In total, 26 runners (9 males, 8 amenorrheic females, and 9 menstruating females) and 20 controls (10 males and 10 menstruating females) participated in this study (Table 1). Seven menstruating females and all female controls completed the tests during both menstrual phases. Two participants (an amenorrheic runner and a female control) had missing polysomnographic sleep recordings. Patients with current medical conditions, metabolic diseases, or smoking habits were not included. The participants were not taking any medications affecting glucose or lipid metabolism, and none had thyroid or heart disease. Runners had career-competitive middle- or long-distance (800–5,000 m) running experience for at least three years. These chronically trained runners were members of a university sports club and generally performed training over 5 days/week. They have an athletic level over the Highly Trained/National Level based on the athlete’s participant classification framework.^56^ All control participants had a sedentary lifestyle and did not engage in regular exercise habits (less than twice per week).

Female controls had low female athlete triad risk scores (<2.0) on the initial screening. The scores were calculated based on the presence of risk factors, assessed through questionnaires, interviews, and body size and DXA measurements. The risk factors included eating disorders, BMI, delayed menarche, the number of menses in 12 months, stress fracture history and bone mineral density, as reported in a previous study.^4^ Menstrual status was determined based on self-reported history and a personal interview; no participant was diagnosed with any gynecological issue. Menstrual status was classified into two categories^56^^,^^57^: amenorrhea and menstruation. Amenorrhea was defined as having less than three menstrual cycles in the last 12 months, with no menstruation occurring for more than three consecutive months. Menstruation was defined as a menstrual cycle of 25–38 days or the occurrence of menstruation in the last 12 months, with more than three menstrual periods.

#### Ethical approval

The study protocols were explained to all participants who provided written informed consent for all procedures. Procedures were conducted in accordance with the Declaration of Helsinki. This study was approved by the Institutional Review Board of the University of Tsukuba, Faculty of Health and Sport Sciences (Ref. Tai 29-29, Tai019-41). The protocol was registered with the University Hospital Medical Information Network Clinical Trials Registry (UMIN000044505).

### Method details

#### Experimental protocol

This study was conducted without modifying the daily lifestyle or training of the participants. Menstruating females were tested twice in the follicular and luteal phases. The participants notified the investigators when menses had started, which was considered day 1 of the menstrual cycle. Measurements were taken between days 9 and 13 of the menstrual cycle for the late follicular phase (10.6 ± 1.0 days) and days 21–24 for the luteal phase (22.4 ± 0.9 days) to measure the physical activity during a week with free-living immediately before the day of EE measurement.

The study was scheduled for eight consecutive days (Figure S3). One week before measuring the metabolic rate, the participants maintained a regular sleep/wake schedule. Daily diet and physical activity were recorded for 7 days, including the day of the experiment. The participants were instructed to refrain from caffeine-containing beverages and alcohol for 3 days before the experiments, and all menstruating females were instructed to consume the same diet for the last meal before both EE measurements. Participants visited our laboratory in the evening on day 7. On the measurement day, the runners did not train to avoid any effects on energy metabolism measurements. The participants ate dinner 5 h before their habitual bedtime to minimize the effects of diet-induced thermogenesis on SMR and OMR.^58^ The dinner consumed before measuring EE was general Japanese food, and menstruating females were asked to have almost the same dinner on the twice EE measurements. Participants entered the whole-room indirect calorimeter after wearing all sensors and voiding urine. They were instructed to maintain a sitting posture until their habitual bedtime (23:00 or 00:00). The sleeping time was 8 h, and the participants were woken by calling out over the intercom leading into the chamber and turning on the lights after 8 h. mSMR was calculated as the mean of the lowest EE observed during three consecutive hours within 8 h sleep,^11^^,^^59^ and mOMR was calculated as the mean EE of entire 8 h sleep.^11^^,^^59^ Subsequently, mRMR was measured in a resting position on the bed for 30 min after a fasting period of at least 13.5 h to avoid diet-induced thermogenesis. To ensure that the mRMR was calculated during the stable supine time, we excluded the first and last 5 min of the supine duration.

#### Measurements

##### Body size and composition

Height and body weight were measured early in the morning using a stadiometer and digital scale in the fasted state after voiding urine and leaving the metabolic chamber. Body composition was measured using bioelectrical impedance analysis (TANITA, BC-118E) during the morning fasting period.

##### Energy metabolism

The room capacity was 14.49 m^3^ (Fuji Medical Science Co., Ltd., Chiba, Japan).^60^ The metabolic chamber was equipped with a bed, desk, chair, and toilet. The airflow in the chamber was rigidly controlled and ventilated at a rate of 80 or 110 L/min. The temperature and relative humidity of the chambers were set to 25°C and 50–55% relative humidity, respectively. Oxygen consumption (VO_2_) and carbon dioxide production (VCO_2_) in outgoing air from the metabolic chamber were measured using an online process mass spectrometer (VG Prima δB; Thermo Electron, Winsford, UK). The accuracy of mass spectrometry, defined as the standard deviation for the continuous measurement of the calibration gas mixture (15% O_2_, 5% CO_2_), was <0.002% for O_2_ and CO_2_. The gas analyzer was calibrated monthly using four gas-type calibration bottles. The time courses for VO_2_ and VCO_2_ every 60 s were calculated using the deconvolution algorithm^61^; EE and the respiratory exchange ratio were calculated from VO_2_, VCO_2_, and urinary nitrogen excretion measured using the Kjeldahl method, which was assumed to be constant during calorimetry.^62^^,^^63^

The intra-individual coefficient of variation and standard deviation (SD) using a metabolic chamber with 12 participants (9 males and 3 females, age: 21 ± 2 years, BMI: 20.4 ± 3.0 kg/m^2^) for mSMR, mOMR, and mRMR were 3.3, 2.6, and 3.8%, and 61, 52, and 72 kcal, respectively. The intra-individual coefficient of variation and SD were similar to those previously reported.^64^^,^^65^

##### Determination of the measured to estimated metabolic rate ratio

The measured RMR was compared with the estimated RMR. The RMR ratio depends on the accuracy of the mRMR and the selected RMR estimation equation.^9^^,^^66^ The prediction equation used to estimate RMR has been previously reported,^67^^,^^68^ and cutoff values have been established.^9^^,^^69^ The cutoff value for energy deficiency was 0.94 for the RMR ratio, developed explicitly for menstrual disturbances.^9^^,^^70^ The estimated equation for RMR is as follows:

Estimated RMR (kJ/day) = (brain mass × 1004 + skeletal muscle mass × 54 + bone mass × 10 + adipose tissue mass × 19 + residual mass × 180)

SMR and OMR, which are more stable and reproducible than RMR, were used.^11^^,^^12^ The estimated SMR^13^ and OMR^11^^,^^71^ were calculated using each estimate equation developed for healthy Japanese individuals as follows:

Estimated SMR (kJ/day) = 77 × FFM +1815

Estimated OMR (kJ/day) = (0.0812 × FFM +0.0213 × fat mass (FM) – 0.2125 × sex +1.8175) × 1000

(sex is 1 for males and 2 for females)

The estimated RMR, SMR, and OMR were calculated using body composition measured by whole-body dual-energy X-ray absorptiometry (DXA; Horizon, Hologic, QDR System Software Version 12.6). Certified radiation technicians performed all DXA measurements according to the research ethics in Japan.

##### Sleep recording and EE between sleep stages

Sleep was recorded polysomnographically (PSG-1100; Nihon Kohden, Tokyo, Japan). Six electroencephalograms, two electrooculograms, and one submental electromyogram were recorded.^72^ Sleep stages were scored manually every 30 s during REM sleep, N1, N2, and SWS by a registered polysomnographic technologist according to the American Academy of Sleep Medicine criteria.^72^ EE between sleep stages was employed only when the same sleep stage continued for at least 60 s.

##### Physical activity in free-living conditions

Active EE was estimated based on the metabolic equivalents monitored using a tri-axial accelerometer (Active style Pro HJA-750C; OMRON Healthcare, Japan) on the waist with reports from participants and mRMR, whereas PAL was calculated based on a previous study as follows: PAL = total EE/mRMR.^73^

### Quantification and statistical analysis

Data are presented as the mean ± SD. All analyses were performed using IBM SPSS 28.0 for Windows. The statistical significance level was set at a *p*-value <0.05 for all analyses. We confirmed normality using Shapiro–Wilk test and homogeneity of variance assumptions using Levene test in all variables. The relationships between mSMR, mOMR, and mRMR were examined using Pearson’s correlations. The characteristics of participants, ratios of measured-to-predicted EE, and sleep architectures were analyzed using one-way analysis of variance (ANOVA) between five groups (amenorrheic runner, female runner, female control in the follicular phase, male runner, and male control; Tables 1, 2, and 3). We also used Student’s paired *t*-test to compare mRMR, core body temperature, PAL, ratios of measured-to-predicted EE, and sleep architectures between the follicular and luteal phases (Tables 1, 2, and 3). Comparisons were made between the two groups in nine combinations to address the research questions regarding EE during sleep and sleep architecture, as shown in Figure S4. The inter-individual differences in EE at each sleep stage, mSMR, and mOMR between the groups were analyzed using analysis of covariance (ANCOVA) with FFM and FM as covariates.^11^^,^^13^ The interaction for the time course of EE during sleep was analyzed using two-way ANCOVA for mixed time course designs, adjusted by FFM and FM as covariates (Figures 1, 3, and 4). Post-hoc analysis was used for *t*-tests between the groups. When compared based on the menstrual cycle, the interaction for the time course of EE during sleep was analyzed using two-way ANOVA for within-subject designs (Figure 2). Differences in mSMR, mOMR and sleep architecture between the follicular and luteal phases were analyzed using Student’s paired *t*-tests.

We compared the two groups across 16 points during sleep (Figures 1, 2, 3, 4, and S4). The sample size was calculated using a repeated measures ANOVA with a within-between interaction in G∗Power (G∗Power 3.1.9.7, Universität Kiel, Germany), with an effect size f of 0.4 based on a previous study,^68^ an α of 0.05, a power of 0.95, a correlation among the repeated measures of 0.5, and a no-sphericity correction of 1.0. The total sample size was calculated as 8, with 4 participants per group.
