# Supplementary material for: Energy expenditure and slow-wave sleep in runners: Focusing on reproductive function, chronic training, and sex
Source: iScience. 2025 Jan 3;28(2):111717. doi: 10.1016/j.isci.2024.111717 (PMC11787595; doi:10.1016/j.isci.2024.111717)
Supplement: Document S1. Figures S1–S4 and Tables S1 and S2 [file mmc1.pdf]

iScience, Volume *28*

Supplemental information

Energy expenditure and slow-wave sleep in runners: Focusing on reproductive function, chronic training, and sex

Akiko Uchizawa, Haruka Osumi, Simeng Zhang, Katsuhiko Yajima, Airi Funayama, Emi Kondo, Yoko Suzuki, Yoshiaki Tanaka, Insung Park, Yasushi Enomoto, Naomi Omi, Kumpei Tokuyama, and Hiroyuki Sagayama

SUPPLEMENTAL MATERIALS

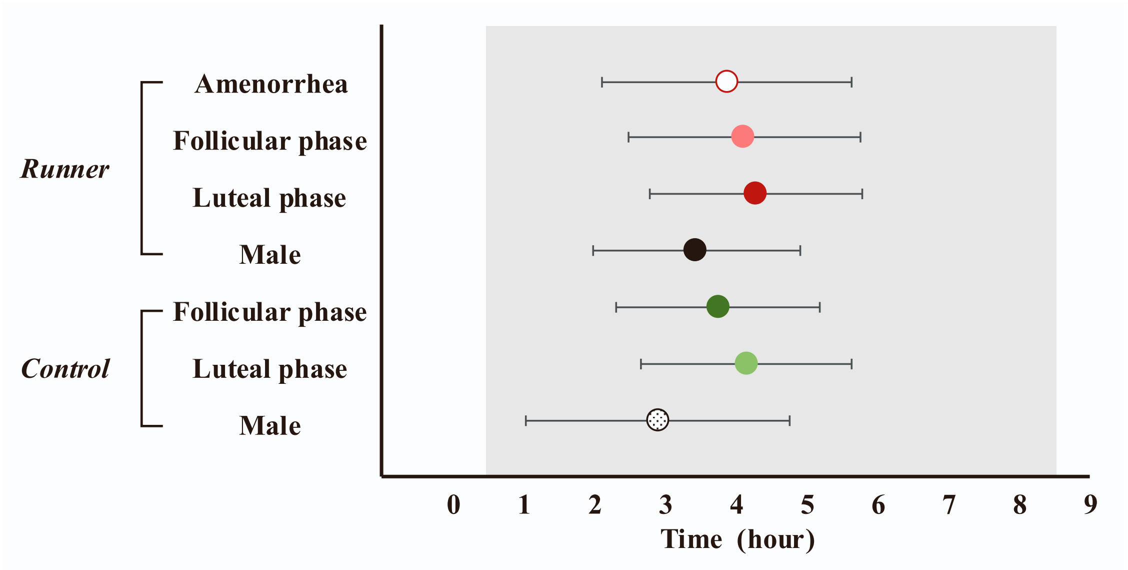

Fig. S1. Start time for measured sleeping metabolic rate (SMR).

The average start time for the measured SMR is shown.

The start time for the measured SMR was analyzed using one-way analysis of variance among five groups (amenorrheic runner, female runner in the follicular phase, female control in the follicular phase, male runner, and male control). Intra-individual changes were analyzed using Student's paired *t*-test between menstrual phases. The start time for the measured SMR is shown as the mean ± SD.

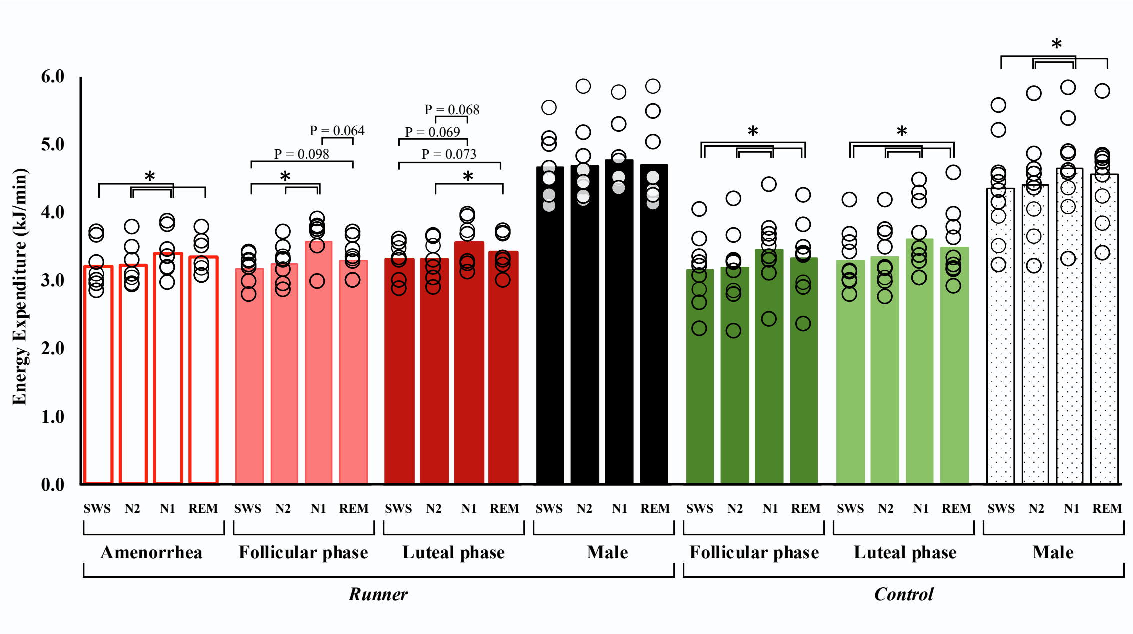

Fig. S2. Mean energy expenditure (EE) in different sleep stages.

The values are unadjusted as the results show intra-individual EE differences for each sleep stage. The circles indicate the individual EE of each sleep stage. The intra-individual EE between sleep stages was analyzed using one-way repeated measures analysis of variance with Bonferroni's post-hoc tests. $^{*}P < 0.05$. SWS, slow-wave sleep; N2, non-rapid eye movement sleep stage 2; N1, non-rapid eye movement sleep stage 1; REM, rapid eye movement.

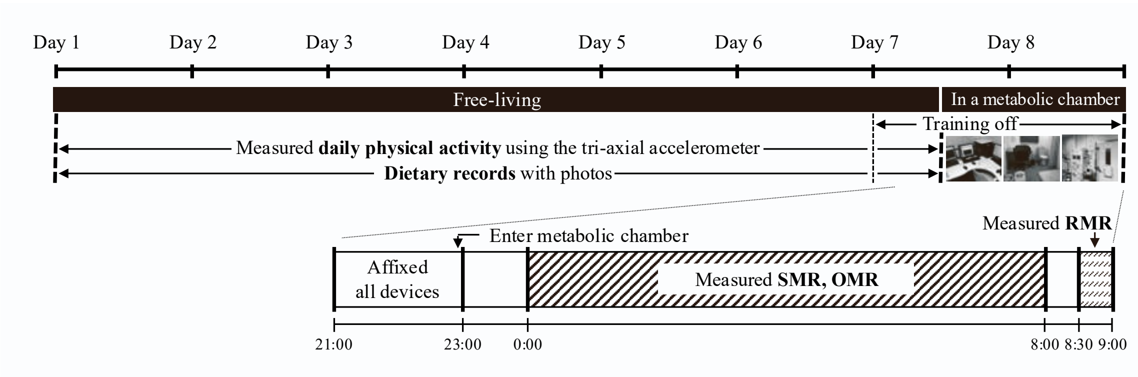

Fig. S3. Experimental protocol of the STAR methods (section Experimental protocol)

SMR, sleeping metabolic rate; OMR, overnight metabolic rate; RMR, resting metabolic rate.

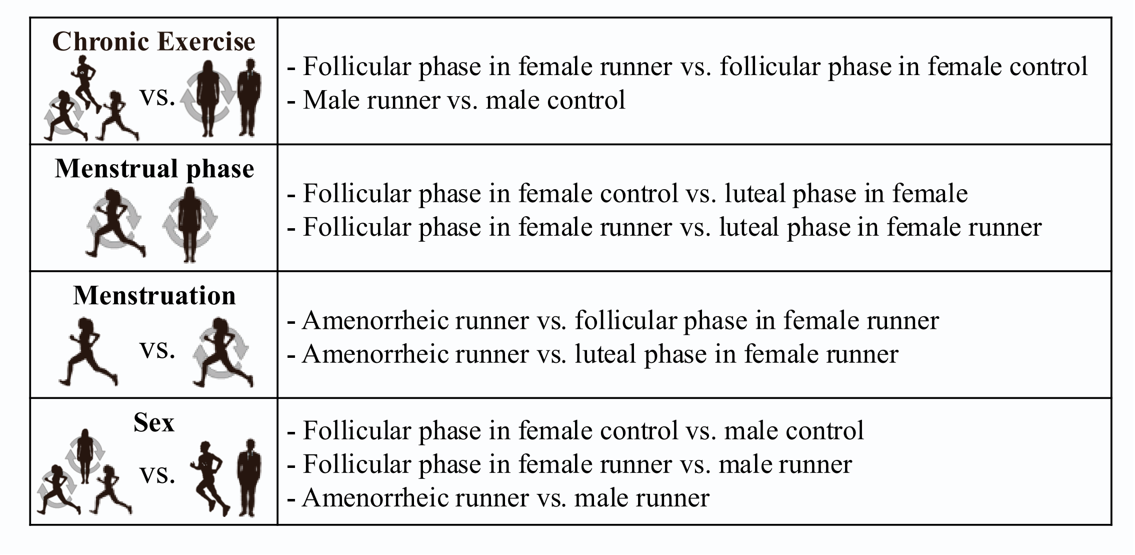

Fig. S4. Comparison combinations, related to STAR methods (section Statistical analysis).

Table S1(a). Inter-individual comparison of the continuous duration of rapid-eye-movement sleep and slow-wave sleep.

	Runner			***Control***	
	**Female**		**Male (n = 9)**	**Female**	**Male (n = 10)**
	**Amenorrhea (n = 7)**	**Follicular phase (n = 9)**		**Follicular phase (n = 9)**	
**REM, min**	22.4 ± 5.4	35.0 ± 11.4*	25.9 ± 9.5	25.4 ± 10.8	21.3 ± 6.1
**SWS, min**	47.0 ± 12.6	40.6 ± 15.0	48.0 ± 13.9*	48.0 ± 19.1*	27.5 ± 12.2

Continuous durations were analyzed using a one-way analysis of variance.

*$P < 0.05$ vs. male of control. REM, rapid-eye-movement sleep; SWS, slow-wave sleep.

Table S1(b). Intra-individual comparison of the continuous duration of rapid-eye-movement sleep and slow-wave sleep in menstruating females.

	Runner **(n = 7)**		**P value**	***Control*** **(n = 9)**		**P value**
	**Follicular phase**	**Luteal phase**		**Follicular phase**	**Luteal phase**	
**REM, min**	32.7 ± 12.0	24.7 ± 5.4	0.086	25.4 ± 10.8	22.9 ± 12.0	0.102
**SWS, min**	37.6 ± 15.8	38.3 ± 14.5	0.891	48.0 ± 19.1	38.3 ± 18.4	0.587

Intra-individual continuous durations were analyzed using a Student's paired t-test.

REM, rapid-eye-movement sleep; SWS, slow-wave sleep.

Table S2(a). Multiple regression model analyzing inter-individual variability in energy expenditure.

	Sleeping metabolic rate			Overnight metabolic rate			Resting metabolic rate		
	**β**	**standard error**	***P***	**β**	**standard error**	***P***	**β**	**standard error**	***P***
Intercept		402.148	0.421		415.163	0.406		480.828	0.168
Fat-free mass	0.884	8.651	< 0.01	0.885	8.931	< 0.01	0.859	10.344	< 0.01
group			0.569			0.576			0.322
Fat mass			0.976			0.983			0.717

F-value for entry and removal of variables was set at 2.00 and 0.15, respectively. Stepwise multiple regression analysis was used with fat mass, fat-free mass, and group as the examination predictors. *n* = 46 (8 amenorrheic runners, 9 female runners in follicular phase, 10 female controls in follicular phase, 9 male runners, 10 male controls). Fat mass and group were not entered.

Table S2(b). Multiple regression model analyzing intra-individual variability in energy expenditure among menstruating females.

	Sleeping metabolic rate			Overnight metabolic rate			Resting metabolic rate		
	**β**	**standard error**	***P***	**β**	**standard error**	***P***	**β**	**standard error**	***P***
Intercept		808.871	0.443		775.093	0.186		823.428	< 0.05
Fat-free mass	0.657	20.377	< 0.01	0.578	21.101	< 0.01	0.400	22.417	< 0.01
Fat mass	-	-	-	0.218	15.734	0.134	0.454	16.715	< 0.01
Menstrual phase			0.268			0.258			0.572
Fat mass			0.180			-			-

F-value for entry and removal of variables was set at 2.00 and 0.15, respectively. Stepwise multiple regression analysis was used with fat mass, fat-free mass, and menstrual phase as the examination predictors. *n* = 17 (7 female runners with menstrual cycle, 10 female controls). Fat mass was not entered for sleeping metabolic rate. The menstrual phase was not entered for all multiple regression model.
